# Pim Kinase Inhibition Disrupts CXCR4 Signalling in Megakaryocytes and Platelets by Reducing Receptor Availability at the Surface

**DOI:** 10.3390/ijms25147606

**Published:** 2024-07-11

**Authors:** Sophie H. Nock, Maria R. Blanco-Lopez, Chloe Stephenson-Deakin, Sarah Jones, Amanda J. Unsworth

**Affiliations:** 1 Department of Life Sciences, Faculty of Science and Engineering, Manchester Metropolitan University, Manchester M1 5GD, UK; 2 Discovery and Translational Science Department, Leeds Institute of Cardiovascular and Metabolic Medicine, University of Leeds, Leeds LS2 3AA, UK

**Keywords:** Pim kinase, CXCR4, thrombopoiesis, signalling, GPCR, CXCL12/SDF1α, platelets, megakaryocytes

## Abstract

A key step in platelet production is the migration of megakaryocytes to the vascular sinusoids within the bone marrow. This homing is mediated by the chemokine CXCL12 and its receptor CXCR4. CXCR4 is also a positive regulator of platelet activation and thrombosis. Pim-1 kinase has been shown to regulate CXCR4 signalling in other cell types, and we have previously described how Pim kinase inhibitors attenuate platelet aggregation to CXCL12. However, the mechanism by which Pim-1 regulates CXCR4 signalling in platelets and megakaryocytes has yet to be elucidated. Using human platelets, murine bone marrow-derived megakaryocytes, and the megakaryocyte cell line MEG-01, we demonstrate that pharmacological Pim kinase inhibition leads to reduced megakaryocyte and platelet function responses to CXCL12, including reduced megakaryocyte migration and platelet granule secretion. Attenuation of CXCL12 signalling was found to be attributed to the reduced surface expression of CXCR4. The decrease in CXCR4 surface levels was found to be mediated by rapid receptor internalisation, in the absence of agonist stimulation. We demonstrate that pharmacological Pim kinase inhibition disrupts megakaryocyte and platelet function by reducing constitutive CXCR4 surface expression, decreasing the number of receptors available for agonist stimulation and signalling. These findings have implications for the development and use of Pim kinase inhibitors for the treatment of conditions associated with elevated circulating levels of CXCL12/SDF1α and increased thrombotic risk.

## 1. Introduction

To initiate platelet production, mature megakaryocytes (MKs) migrate through the bone marrow to the sinusoidal niche [1]. This process has been shown to be partially mediated by the chemokine receptor CXCR4, which is expressed on the MK surface and can induce intracellular signalling responses once bound to its ligand: C-X-C Motif Chemokine Ligand 12 (CXCL12)/Stromal Cell-Derived Factor 1 (SDF1α)/CXCL12 [2,3]. CXCL12 is highly expressed and released by vascular endothelial cells, reticular cells, and bone marrow stromal cells, which encourages MK migration to the sinusoidal niche where proplatelet formation and thrombopoiesis occur [3,4,5]. Loss of CXCL12/CXCR4 signalling and interaction results in a failure of chemotaxis and reduced platelet production [6,7,8]. CXCR4 is also a positive regulator of platelet activation and thrombosis. Platelets contain CXCL12 in their alpha granules, which is released following platelet activation and degranulation [9,10,11]. CXCL12 signalling via CXCR4 can enhance other agonist-mediated platelet responses, and CXCL12 stimulation can drive Ca^2+^ mobilisation and degranulation, and integrin activation and, aggregation [10,11,12,13].

Thromboinflammation refers to the activation of platelets and immune cells to promote thrombosis either in the presence or absence of a pathogen. Chemokines, including CXC12, have been strongly implicated in thromboinflammation [14]. Many systemic inflammatory diseases such as rheumatoid arthritis [15], systemic lupus erythematous [16], and familial Mediterranean fever [17] have upregulated CXCR4 and/or increased circulating plasma levels of CXCL12, and these conditions also carry a predisposition to thrombosis [18]. 

CXCR4 is a seven-transmembrane-spanning G-protein-coupled receptor (352 amino acids, 48 kDa) [19,20]. When CXCL12 binds to CXCR4, it triggers Gαi G-protein-coupled signal transduction pathways, including extracellular signal-regulated kinase-/mitogen-activated protein kinase (ERK/MAPK) cascade activation and intracellular calcium flux-regulating chemotaxis, transcription, and cell survival [11,21,22]. As with many GPCRs, CXCR4 signalling is mediated by desensitisation and receptor internalisation following ligand binding, which is mediated by GPCR kinase (GRK)-dependent phosphorylation and interaction with β-arrestin [23,24], although CXCR4 is also known to undergo constitutive internalisation in the absence of agonist, via dynamin and protein kinase C (PKC)-dependent processes [7]. 

Pim (Proviral Integration Site for Moloney Murine Leukaemia Virus) kinases include three constitutively active serine/threonine kinases, Pim-1, Pim-2, and Pim-3, that are regulated predominantly by transcription and translation downstream of JAK–STAT signalling pathways and proteasomal degradation [25,26]. Studies investigating the role of Pim kinase in haemopoietic cells demonstrated that mice deficient in Pim-1 have defects in haematopoietic stem cell (HSC) homing to the bone marrow caused by a downregulation of CXCR4 surface expression [27]. Studies using primary acute myeloid leukaemia blasts and in vitro cell lines have further elucidated that Pim kinase inhibition using pharmacological inhibitors also downregulates CXCR4 on the cell surface, which leads to a reduction in CXCL12/CXCR4 signalling and cell migration [28,29]. 

Triple knock-out mice deficient in all three Pim kinase isoforms have altered haematopoiesis [30]. However, in clinical trials for Pim kinase inhibitors targeting all three isoforms there are conflicting reports of thrombocytopenia [31], with some studies reporting thrombocytopenia [32], whilst others not [33]. There is some dispute as to whether the cause of the disruption of the platelet count associated with global Pim depletion is an alteration of thrombopoiesis, a process mediated by the CXCL12/CXCR4 signalling axis, and this has yet to be investigated. We have previously shown the anti-platelet properties of Pim kinase inhibitors, demonstrating attenuated platelet aggregation responses to CXCL12 in addition to thromboxane A2 in the presence of Pim kinase inhibition [34]. The role of Pim kinase in megakaryocytes, and the mechanism by which Pim kinase regulates CXCR4 signalling in platelets and megakaryocytes remains to be explained.

Herein, we describe how Pim kinase inhibition alters CXCR4 signalling in megakaryocytes and platelets via receptor internalisation, independent of agonist stimulation. Inhibition of megakaryocyte and platelet CXCR4 using pharmacological inhibition results in decreased surface expression of CXCR4, attenuated signalling following stimulation with CXCL12, and reduced megakaryocyte migration to CXCL12. 

## 2. Results

### 2.1. Pim Kinase Regulates CXCR4 Signalling Events in Megakaryocytes

CXCL12/CXCR4 signalling is an important signalling axis that facilitates platelet production and thrombopoiesis [4]. Previous studies using leukemic cell lines have demonstrated reduced lymphoid cell migration to CXCL12 following the genetic deletion or pharmacological inhibition of Pim kinase [28,29]. The role of Pim kinase in megakaryocyte function and migration was therefore investigated.

**Figure 1:**
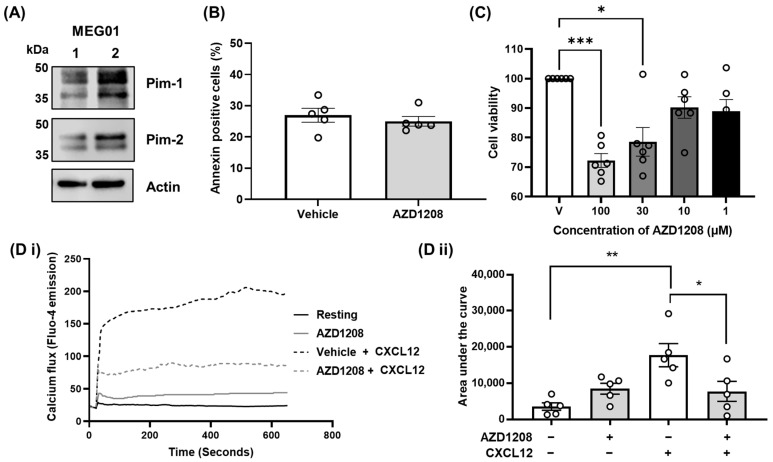
MEG01s express Pim kinase, and its inhibition attenuates signalling responses to CXCL12. (**A**) MEG01 cells were lysed with a RIPA buffer and analysed via SDS-PAGE followed by western blotting using an anti-Pim-1 and anti-Pim-2 antibody. Actin was included as a loading control. Representative blots of MEG01 lysates were used at Passage 10. (**B**) MEG01 cells were incubated with a high concentration of AZD1208 (100 µM) for 2 h before cells were stained with Annexin V to assess for cell viability using flow cytometry. Cells were gated by FSC and SSC, and 5000 events were recorded. The percentage of Annexin V positive cells was gated on an unstained sample. (**C**) MEG-01 cells were incubated with increasing concentrations of AZD1208 (1, 10, 30, and 100 µM) or the vehicle control (0.1% DMSO) for 24 h before proliferation and viability were assessed using an MTS assay according to manufacturers’ instructions. Normalised data are shown with statistical testing performed on non-normalised data. (**D**) MEG01 cells were loaded with Fluo-4 and Pluronic acid and preincubated with AZD1208 (10 µM) or the vehicle control for 90 min. Calcium flux was induced by stimulation with 200 ng/mL CXCL12, and responses were monitored for 10 min using a GloMax® Explorer plate reader (Promega, Southampton, UK). (**D i**) Representative trace of *n* = 5 experiments, **(D ii**) calcium mobilisation data quantified and expressed as area under the curve. Statistical testing was performed using a two-way ANOVA. All quantified data points are shown (◦), *n* ≥ 4, error bars are mean ± SEM, * *p* ≤ 0.05, ** *p* ≤ 0.01. *** *p* ≤ 0.001.

To explore the mechanism of platelet and megakaryocyte CXCR4 receptor regulation via Pim kinase, signalling studies using the megakaryocyte-like cell line MEG-01 [35] were performed. First, the protein expression of Pim-1 and Pim-2 isoforms was confirmed in MEG-01 cells using western blotting (Figure 1A). We have previously used the Pim kinase inhibitor AZD1208 (up to 10 µM) for investigations using washed platelets [34] at concentrations known to be reached in vivo (≥10 µM) [36], and demonstrated that other Pim kinase inhibitors elicit similar effects on platelet functional responses [34]. Pim-2 kinase has been shown previously to play important roles in cell survival [29]. Therefore, cell viability tests (Annexin V binding) and proliferation assays (MTS) were performed using MEG-01 cells to assess the toxicity of pharmacological Pim kinase inhibitor treatment under the conditions used (10 µM for 24 h) (Figure 1B,C). Treatment with 10 µM AZD1208 for 24 h [34] did not increase phosphatidylserine exposure, a marker of cell death (Figure 1B), and did not alter cell proliferation and viability (Figure 1C), although higher concentrations (30, 100 µM) did reduce cell proliferation and viability.

To determine whether Pim kinase is involved in megakaryocyte cell responses following CXCR4 stimulation, the effect of Pim kinase inhibitor treatment on calcium mobilisation in MEG-01 cells was determined following stimulation by CXCL12 (200 ng/mL). Acute treatment with Pim kinase inhibitor AZD1208 (10 µM) caused an attenuation of CXCL12-induced calcium mobilisation, indicating reduced CXCR4 receptor signalling responses in MEG-01 cells (Figure 1D). 

### 2.2. Pim Kinase Positively Regulates Megakaryocyte Homing and Migration to CXCL12

**Figure 2:**
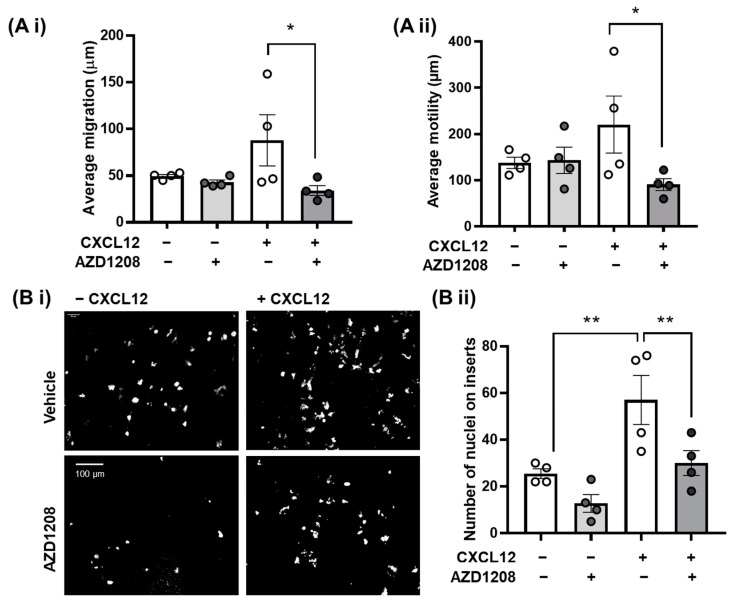
Pim kinase inhibition attenuates megakaryocyte migration to CXCL12. (**A**) MEG01 cells or (**B**) murine bone marrow-derived mature megakaryocytes were incubated with AZD1208 (10 µM) or the vehicle (0.1% DMSO) control for 30 min, prior to the addition of 100 ng/mL CXCL12. (**A i**) MEG01 cell migration and (**A ii**) motility in the presence of CXCL12 was recorded and measured over time (24 h) using a Holomonitor Live Cell Imaging system. (**B**) Primary megakaryocytes were added to transwell membrane inserts, and migration towards CXCL12 (300 ng/mL) was measured for 5 h using DAPI, counting the number of cells that passed through the transwell insert using a Celena Logos and 4x objective lens. (**B i**) Representative images and (**B ii**) quantified data are shown. (**A**,**B**) All quantified data points are shown (◦ white circles = vehicle, grey circles = + AZD1208)), *n* = 4, error bars are mean ± SEM, * *p* ≤ 0.05, ** *p* ≤ 0.01.

In addition to the inhibition of CXCR4 signalling events, treatment with Pim kinase inhibitors also attenuated the ability of MEG-01s to migrate towards CXCL12, represented by a significant reduction in average cell motility and migration over a 24 h period following treatment with AZD1208 (Figure 2A).

To confirm that our observations in MEG01 cells are translatable to primary megakaryocytes, migration to CXCL12 was investigated using mature murine bone marrow-derived megakaryocytes. Supporting the results in MEG-01 cells and confirming an inhibitory effect of Pim kinase inhibition on megakaryocyte migration, CXCR4-/CXCL12-mediated megakaryocyte migration across a CXCL12 transwell gradient was attenuated in the presence of the Pim kinase inhibitor AZD1208 (10 µM) (Figure 2B). As megakaryocyte migration to the sinusoidal niche, mediated by CXCL12 signalling, is a key step in thrombopoiesis, these findings may explain observations of thrombocytopenia as an adverse effect in clinical trials investigating the potential use of Pim kinase inhibitors as cancer treatments [36,37,38,39].

### 2.3. Pim Kinase Inhibition Reduces CXCR4 Surface Expression Levels in Megakaryocytes

**Figure 3:**
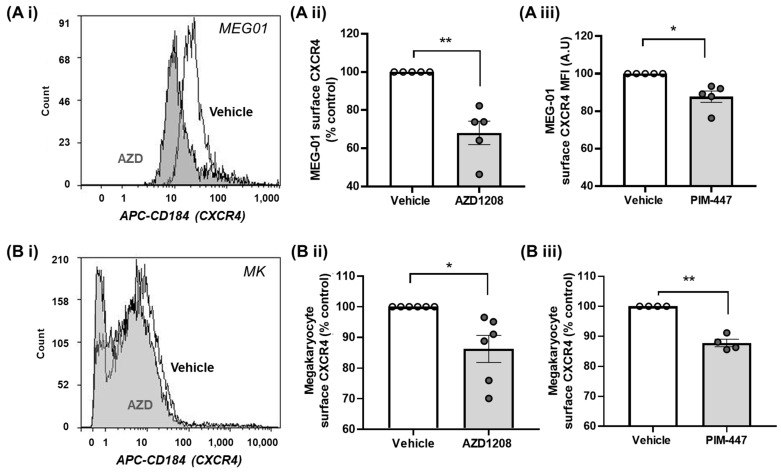
Pim kinase inhibition reduces surface expression levels of CXCR4 in megakaryocytes. (**A**) MEG01 cells and (**B**) mature bone marrow-derived primary megakaryocytes (MK) were treated with (**A i,ii**) 10 µM AZD1208 or (**A iii**) PIM-447 for 30 min before staining for CD41 and CXCR4 was carried out on ice for 30 min. Cells were washed and run on a flow cytometer, where 10,000 CD41+ve events were gated, CXCR4 positive events were gated, and the median fluorescence intensity of CXCR4 was determined. (**B i**) Representative histogram with AZD1208-treated samples shown in grey and vehicle-treated samples in clear. Statistical testing was performed using a Student’s *t*-test or a one-way ANOVA. (**B ii,iii**) All quantified data points are shown (◦ white circles = vehicle, grey circles = + treatment), *n* ≥ 5, error bars are mean ± SEM, * *p* ≤ 0.05, ** *p* ≤ 0.01.

Reduced responses to CXCL12 following Pim kinase inhibition in other cell types has been attributed to the reduced surface expression of CXCR4 [28,29]. MEG01 cells and primary murine megakaryocytes were therefore treated with Pim kinase inhibitors AZD1208 (10 µM), PIM447 (10 µM), or the vehicle control (0.1% DMSO), and CXCR4 surface expression was determined using flow cytometry and an antibody that recognises the extracellular portion of CXCR4. Similar to previous observations in other haematopoietic cell types, and in line with our signalling and functional findings, treatment with either of the Pim kinase inhibitors (AZD1208 or PIM447) resulted in a reduction in CXCR4 surface expression levels in MEG-01 cells (AZD1208: 31.9 ± 6.2, PIM-447: 13.3 ± 3.0% inhibition) and a 15% reduction in the number of primary megakaryocytes expressing CXCR4 compared to the vehicle-treated (0.1% DMSO) controls (AZD1208: 19.2 ± 6.4, PIM-447 13.3 ± 1.2% inhibition) (Figure 3B). This demonstrates that Pim kinase activity is required for the constitutive surface expression of CXCR4 in megakaryocytes. 

### 2.4. Megakaryocyte Cells Attempt to Overcome Pim Kinase Inhibition by Increasing Pim-1 and CXCR4 Expression

**Figure 4:**
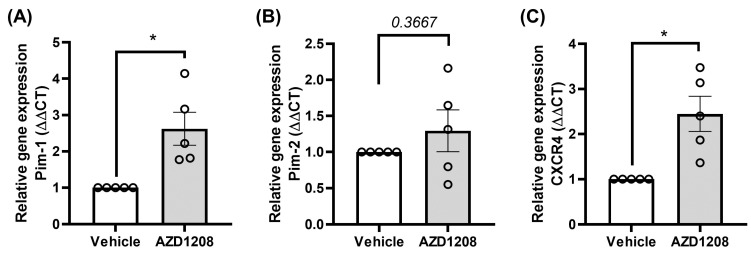
Megakaryocyte-like cells increase Pim-1 and CXCR4 gene expression to compensate for Pim kinase inhibition. MEG-01 cells were treated with 10 µM AZD1208 for 24 h and RNA was extracted using a Qiagen kit, converted to cDNA, and analysed via qPCR for mRNA expression of (**A**) Pim-1, (**B**) Pim-2, and (**C**) CXCR4. Statistical testing was performed using a Student’s *t*-test, *n* = 4, all quantified data points are shown (◦), error bars are mean ± SEM, * *p* ≤ 0.05.

The Pim kinase family are constitutively active enzymes, and their activity is regulated by protein expression and degradation. To determine whether megakaryocytes attempt to compensate for the reduction in cellular responses caused by the Pim kinase inhibitor though an upregulation of the Pim kinase isoforms or CXCR4 expression, an RT-qPCR analysis of Pim-1, Pim-2, and CXCR4 mRNA levels was performed on MEG-01 cells, following treatment with the Pim kinase inhibitor. In response to 24 h of treatment with AZD1208 (10 µM), both Pim-1 and CXCR4 were upregulated in MEG-01s (Figure 4). These findings provide some evidence that Pim-1 kinase is likely responsible for the regulation of CXCR4 surface exposure, in line with observations made in other cell types [28,29,40], and they demonstrate that MEG-01 cells attempt to overcome pan-Pim kinase inhibition through an increased expression of Pim-1 and CXCR4.

### 2.5. Pim Kinase Inhibition Reduces Platelet CXCR4 Responses via Receptor Internalisation

**Figure 5:**
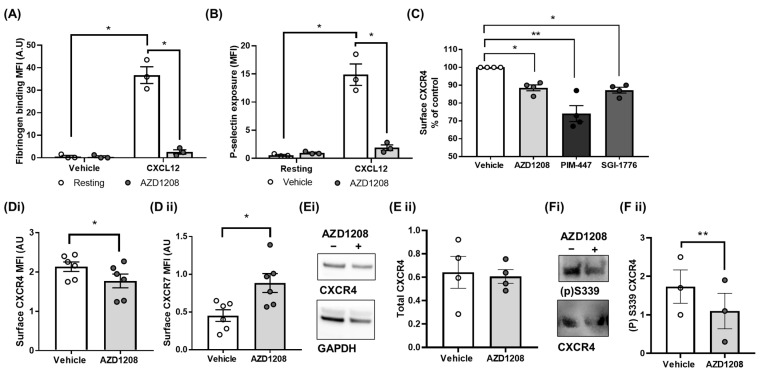
Pim kinase inhibition reduces CXCR4 receptor platelet responses via receptor internalisation. PRP was incubated with AZD1208 (100 µM) or the vehicle (0.1% DMSO) control for 30 min prior to (**A**,**B**) stimulation with CXCL12 (1 ug/mL) for 20 min in the presence of (**A**) anti-fibrinogen (FITC conjugated) and (**B**) anti-CD62P (PECy5 conjugated) antibodies, and median fluorescence intensity was determined using flow cytometry. (**C**) PRP was incubated with AZD1208 (100 µM), PIM-447 (100 µM), SGI-1776 (100 µM), or the vehicle (0.1% DMSO) control for 30 min prior to incubation with an anti-CXCR4 (CD184) antibody that recognises the extracellular portion of CXCR4 for 20 min at room temperature. (**D**) PRP was incubated with AZD1208 (100 µM) or the vehicle (0.1% DMSO) control for 30 min prior to incubation with an (**D i**) anti-CXCR4 (CD184) antibody or (**D ii**) anti-CXCR7 antibody that recognises the extracellular portion of CXCR7. Flow cytometry was performed using a Miltenyi MACsQuant flow cytometer, platelets were gated using FSC and SSC profiles, and 10,000 events were counted. Analysis was performed using Flowlogic, and the positive percentage was gated on vehicle IgG-treated samples. Median fluorescence intensity was obtained and plotted with statistical testing performed using a two-way ANOVA. (**E**,**F**) Washed platelets treated with or without AZD1208 were lysed with a Laemelli buffer before being subjected to SDS-PAGE and western blotting with (**E**) a total CXCR4 antibody and GAPDH antibody, or (**F**) a S339 phospho-site specific CXCR4 antibody and a total CXCR4 antibody, and the densitometry analysis of the blot image performed. (**E**,**F i**) representative blots and (**E**,**F ii**) quantified data are shown. Statistical testing was performed using a one-way SIS, carried out using ImageJ (Fiji, version 2.15.1). All quantified data points are shown (◦ white circles = vehicle, grey circles = + treatment), *n* ≥ 4, error bars are mean ± SEM, * *p* ≤ 0.05, ** *p* ≤ 0.01.

Having identified that Pim kinase inhibition attenuates megakaryocyte CXCR4 signalling and functional responses via the regulation of receptor surface expression, we next evaluated whether Pim kinase pharmacological inhibition with AZD1208 has the same effect on CXCR4 biology in platelets, using platelet-rich plasma. We have previously described reduced platelet aggregation to CXCL12 stimulation following treatment with Pim kinase inhibitors [34]. To overcome plasma binding effects, as validated previously [34], ten-fold higher concentrations of Pim kinase inhibitors were used (100 µM). In further support of Pim kinase activity being required for CXCL12-mediated platelet responses, flow cytometry experiments showed a reduction in CXCL12-mediatedαIIbβ3 integrin activation and alpha granule secretion, with reduced platelet fibrinogen binding (Figure 5A) and surface exposure of P-selectin (Figure 5B), observed following acute treatment with Pim kinase inhibitor AZD1208. To determine whether the pharmacological inhibition of Pim kinase also alters surface expression levels of platelet CXCR4, the effect of Pim kinase inhibitors AZD1208, PIM447, and SGI-1776 on platelet CXCR4 surface expression levels was determined using flow cytometry (Figure 5C).

As anticipated, platelets treated with the Pim kinase inhibitors (AZD1208, PIM447, or SGI-1776) showed reduced surface expression levels of CXCR4 (Figure 5C) compared to the vehicle-treated control (AZD1208: 12.1 ± 1.6, PIM-447: 15.9 ± 4.5, and SGI-1776: 13.1 ± 1.6% inhibition). Interestingly, other studies investigating CXCR4 biology in platelets have previously shown that CXCR4 surface expression levels have a reciprocal relationship with CXCR7 in platelets [12,13], with the decreased surface expression of CXCR4 associated with the increased expression of CXCR7. In line with these findings, we also observed an increase in CXCR7 surface levels following treatment with Pim kinase inhibitor AZD1208, as CXCR4 surface levels decreased (Figure 5D). In support of reduced CXCR4 surface expression levels resulting from increased internalisation and not degradation, an analysis of the total CXCR4 levels via western blotting analysis confirmed that the total protein levels of the receptor remain unchanged in platelets following Pim kinase inhibitor treatment (Figure 5E). Similar to observations made in other cell types [28,29], treatment with Pim kinase inhibitor AZD1208 also reduced the phosphorylation of platelet CXCR4 at S339 in basal conditions (Figure 5F). These findings demonstrate that Pim kinase activity is required for the constitutive surface expression of CXCR4 and signalling in platelets.

### 2.6. Pim Kinase Inhibitors Induce Rapid Receptor Internalisation and Prevent Recycling to the Plasma Membrane

CXCR4 is known to undergo both constitutive and agonist-induced internalisation through distinct pathways, which are dynamin- and arrestin-dependent, respectively [41,42]. Our findings so far demonstrate Pim kinase inhibitors can induce the internalisation of CXCR4 in the absence of agonist stimulation. To investigate the mechanism by which CXCR4 internalisation occurs, CXCR4 surface expression in the presence and absence of Pim kinase inhibition was evaluated using platelets treated with different pharmacological inhibitors of endocytosis regulators. 

**Figure 6:**
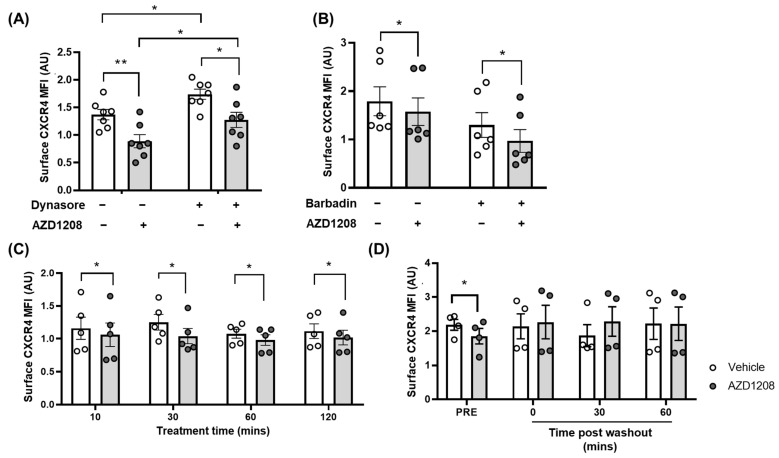
Pim kinase inhibitor-mediated internalisation of CXCR4 is rapid and sustained and is not mediated by dynamin or arrestins. (**A**,**B**) PRP was pre-incubated with (**A**) Dyansore (50 µM) or (**B**) Barbadin (30 µM) prior to 30 min of treatment with AZD1208 (100 µM) or the vehicle. Following the treatments described above, all samples were incubated with an antibody for CXCR4 (CD184) that recognises the extracellular portions of the receptor for 20 min at room temperature. (**C**) PRP was incubated with AZD1208 (100 µM) or the vehicle control (0.1% DMSO) for 10, 30, 60, and 120 min for the analysis of a time course of CXCR4 surface expression. (**D**) PRP was incubated with AZD1208 (100 µM) or the vehicle control (0.1% DMSO) for 30 min prior to wash out, before incubation with the CXCR4 antibody at 0, 30, and 60 min post wash out for the analysis of CXCR4 surface expression recovery. Cells were diluted out before flow cytometry analysis was performed using a Miltenyi MACSQuant flow cytometer. Platelets were gated using FSC and SSC profiles, and 10,000 events were collected. Analysis was performed using Flowlogic, with the percentage of positive events gated on vehicle IgG-treated samples. The median fluorescence intensity was obtained and plotted, with statistical testing performed. All quantified data points are shown (◦ white circles = vehicle, grey circles = + AZD1208), *n* ≥ 5, error bars are mean ± SEM, * *p* ≤ 0.05, ** *p* ≤ 0.01.

The constitutive internalisation of CXCR4 has been previously shown to be a dynamin-dependent process. Therefore, platelets were incubated with Dynasore (50 µM) [43,44], a dynamin inhibitor, prior to treatment with AZD1208. As expected, and in support of constitutive CXCR4 internalisation being a dynamin-dependent process, treatment with Dynasore caused an increase in the platelet surface expression of CXCR4 (Figure 6A). Interestingly however, whilst Dynasore treatment did increase CXCR4 surface expression in AZD1208-treated platelets, CXCR4 surface expression levels were still lower than in vehicle-treated controls in the presence of Dynasore, indicating that Pim kinase inhibitor-mediated reductions in CXCR4 surface expression are not dependent on dynamin-mediated internalisation, and are likely regulated by another pathway. 

We next considered whether Pim kinase inhibition was ‘mimicking’ agonist stimulated internalisation and thereby initiating internalisation via the activation of β-arrestins. The involvement of β-arrestin in CXCR4 surface expression was therefore investigated by pre-treating platelets with Barbadin (30 µM), an inhibitor of β-arrestin/AP2-mediated internalisation at concentrations used previously [45]. In the presence of Barbadin, an AZD1208-mediated reduction in CXCR4 surface expression was not prevented (Figure 6B), indicating that the β-arrestin/AP2 complex is not involved in Pim kinase inhibitor-mediated reductions in CXCR4 receptor surface expression in platelets. 

To further investigate the mechanism by which Pim kinase inhibition reduces CXCR4 surface expression levels, the time taken for receptor internalisation to occur, and the ability of CXCR4 to recycle to the surface was determined following treatment with AZD1208. In platelets, the observed internalisation of platelet CXCR4 is rapid, with a 10% reduction in receptor surface expression observed following only 10 min of inhibitor treatment, and a 20% reduction after 30 min, which is then sustained in the presence of the Pim kinase inhibitor for up to 2 h (Figure 6C), indicating limited recovery of the receptor to the membrane. However, following the washing and removal of AZD1208, a rapid re-externalisation of CXCR4 is observed, with no difference in CXCR4 surface expression levels observed between the treated and the control immediately after washing (Recovery time 0) (Figure 6D), indicating that Pim kinase inhibition may reduce the surface expression of CXCR4 via the inhibition of receptor recycling to the plasma membrane.

## 3. Discussion

Pim kinase-dependent regulation of the CXCR4/CXCL12 signalling axis in haematological cells has been previously described in the context of haematopoietic malignancy [28,40]. This study shows, for the first time, that this regulation also extends to megakaryocytes and platelets. Our results confirm that Pim kinase inhibitors can be used to regulate CXCR4 surface exposure and functional responses using the megakaryocytic cell line (MEG-01), primary mouse megakaryocytes and platelets. In contrast to B cells, where CXCR4 internalisation is ligand (CXCL12)-dependent, Pim kinase inhibition modulates receptor internalisation in megakaryocytes and platelets, causing rapid internalisation under basal conditions, in the absence of an agonist, suggesting that this enzyme is involved in the constitutive regulation of GPCR cycling [20,32]. 

Our findings in platelets suggest the decrease observed in CXCR4 surface levels after pan-Pim kinase inhibition does not occur via established mechanisms of internalisation, as the inhibition of dynamin or β-arrestin/AP2 was not able to prevent Pim kinase inhibitor-mediated reductions in CXCR4 surface expression (Figure 6). Interestingly, in the presence of Pim kinase inhibitors, we also observe no recovery of platelet CXCR4 to the cell surface for up to 2 h, whilst the removal of the inhibitor via washing does lead to the rapid recovery of CXCR4 to the surface, indicating that Pim kinase inhibition may prevent receptor recycling to the plasma membrane. 

CXCR4 activation is crucial for homing, migration, and the retention of various hematopoietic cell types in the bone marrow [46]. Studies of megakaryopoiesis have shown that the upregulation of CXCR4 is associated with the spatial distribution of megakaryocytes within the bone marrow vasculature [4,5]. In addition to supporting megakaryocyte migration to the sinusoidal niche, CXCL12 can also act synergistically with thrombopoietin to enhance megakaryopoiesis and platelet production [47]. As with other GPCRs, CXCR4 receptor function and signalling is strongly dependent on its localisation at the cell surface, where it can bind and be activated by its ligand CXCL12 [21]. 

In addition to its role in bone marrow homing and megakaryopoiesis, it is increasingly evident that CXCR4 is a positive regulator of platelet function and thrombosis. Several recent studies have associated the inhibition of the CXCR4/CXCL12 axis with a reduction in both arterial [48] and venous thrombus formation [14], and there is increasing evidence from population studies linking high CXCL12 levels to an increased thrombotic risk [49,50,51,52]. Several inflammatory conditions associated with reactive thrombocytosis and increased platelet function are also associated with elevated circulating levels of CXCL12/CXCR4 expression [15,16,53,54,55], and CXCR4 has been shown to be implicated in the formation of platelet–neutrophil aggregates [56], which are known to contribute to thromboinflammation. 

Trials and studies investigating the utility of CXCR4 inhibitors for use in haematological malignancies identified that treatment with CXCR4 antagonists, such as Plerixafor, lead to a counter-regulatory mechanism in which the surface expression of CXCR4 is increased [57]. Therefore, for circumstances where targeting CXCR4 signalling may be beneficial, alternative therapies need to be identified. Like other GPCRs, CXCR4 is regulated by desensitisation, internalisation, and degradation [42,58]. CXCR4 can undergo both agonist-dependent and -independent internalisation and surface re-exposure, with previous studies showing that the serine/threonine-rich intracellular C-terminus of CXCR4 plays a key role in both the internalisation and recycling of CXCR4 [59].

Although others have demonstrated that the phosphorylation of S339 in the CXCR4 C-terminal is important in the regulation of CXCR4 by Pim kinase, and we have demonstrated decreased S339 phosphorylation in platelets in the presence of Pim kinase inhibitor AZD1208 [28,29,60], the exact mechanism through which Pim kinase inhibition modulates CXCR4 internalisation still remains to be elucidated. Interestingly, studies of WHIM (Warts, Hypogammaglobulinemia, recurrent bacterial Infections, and Myelokathexis) syndrome indicated that CXCR4 mutants, lacking the terminal 19 residues of the C-terminal tail which includes S339, do not have a decreased surface expression of the receptor [61]. Conversely, these mutants show an increased surface recovery of CXCR4 [62], indicating that the phosphorylation of other serine/threonine residues may be involved in receptor trafficking, in addition to S339 [59]. At this stage, we cannot rule out that Pim kinase may also indirectly regulate the phosphorylation of CXCR4 through another yet unidentified kinase, such as Btk [60], or its surface expression via the recruitment of the receptor for activated protein kinase (RACK) via a different site on the receptor [46]. 

Depending on the cell type and condition, each of the three Pim isoforms have been implicated in the phosphorylation of CXCR4. However, Pim-1 looks to be the most likely to be involved in CXCR4 regulation in the bone marrow environment and haematopoietic cells [28,29,40]. By contrast, Pim-2-deficient bone marrow haematopoietic cells present no defects in the ability to migrate towards CXCL12 compared to WT (wild type) control cells [28]. In support of this, we observed that MEG01 cells attempt to compensate for Pim kinase inhibition by upregulating expression of the Pim-1 isoform but not the Pim-2 (Figure 3). Previous studies in other cell types have demonstrated that Pim kinase deletion or inhibition leads to CXCR4 internalisation over prolonged periods of time [28,29,40]. In contrast, we demonstrated that acute treatment with the Pim kinase inhibitor AZD1208 can induce rapid receptor internalisation in platelets and megakaryocytes, which does not appear to be associated with degradation. 

Several recent studies have associated the inhibition of the CXCR4/CXCL12 axis with a reduction in both arterial [48] and venous thrombus formation [14], with increased circulating levels of CXCL12 and Pim-1 observed in deep vein thrombosis [51]. Furthermore, megakaryocytes expressing high levels of CXCR4 are also enriched for Pim-1. This CXCR4^high^ subset of megakaryocytes also promotes myeloid activation and has immune cell characteristics with the ability of platelet generation [63]. 

Alternative approaches targeting the CXCR4/CXCL12 signalling axis that do not involve direct receptor antagonism may prove to be useful strategies to prevent thromboinflammation. We have previously shown the anti-platelet and anti-thrombotic properties of Pim kinase inhibitors and Pim-1 deletion in in vitro and in vivo models of arterial thrombosis [34]. Our results presented here also provide strong evidence that Pim kinase inhibitors can attenuate CXCR4 downstream signalling and functional responses in megakaryocytes and demonstrate that Pim kinase inhibitors may offer an alternative strategy to target CXCR4-mediated thrombosis.

## 4. Materials and Methods

### 4.1. Materials

CXCL12 and thrombopoietin were purchased from PeproTec (London, UK). AZD1208, PIM-447, dynamin, and Barbadin were purchased from Cambridge Bioscience (Cambridge, UK). MEG-01 cells were obtained from ATCC via LGC Standards, UK Office. Fluo-4AM and CD4 untouched magnetic beads were purchased from Invitrogen/ThermoFisher Scientific (Paisley, UK). The Norgen RNA extraction plus kit was obtained from Geneflow (Lichfield, UK). SYBR green and cDNA synthesis kits were purchased from Qiagen (Manchester, UK). Antibodies were as follows: Pim-1 and Pim-2 antibodies were purchased from Cell Signalling Technology (Leiden, The Netherlands), and CXCR4 was purchased from Proteintech (Manchester, UK). Anti-CD184-APC (CXCR4) and anti-CXCR7 BD Horizon™ BV421 antibodies for flow cytometry were obtained from BD Biosciences (Wokingham, UK). Mouse anti-CD184 and the lineage depletion panel (catalog number: 133307) were obtained from BioLegend UK Limited (London, UK). All other reagents were of chemical grade and purchased from Merck Life Science UK Limited (Gillingham, UK).

### 4.2. Platelet Preparation

Human whole blood was collected by venepuncture from healthy, aspirin-free, consenting volunteers using procedures approved by the Manchester Metropolitan University Ethics Committee. Blood was collected into 3.2% (*v*/*v*) sodium citrate vacutainers before undergoing centrifugation at 100× *g* for 20 min at room temperature to isolate platelet-rich plasma (PRP). For the preparation of the washed platelets, acid citrate dextrose (ACD) (29.9 mM trisodium citrate, 113.8 mM glucose, and 2.9 mM citric acid [pH 6.4]) was added to the PRP (1:8 *v*/*v*) prior to centrifugation at 350× *g* for 20 min to pellet the platelets. Washed platelets were prepared by resuspending the platelet pellet in a modified Tyrode’s HEPES buffer (134 mM NaCl, 0.34 mM Na2HPO4, 2.9 mM KCl, 12 mM NaHCO_3_, 20 mM N-2-hydroxyethylpiperazine-N-2-ethanesulfonic acid, 5 mM glucose, and 1 mM MgCl_2_, pH 7.3) to a platelet count of 4 × 10^8^/mL. For platelet lysates, washed platelets as described above were treated with PGI_2_ (125 ng/mL) before centrifugation at 1400× *g* for 10 min. Platelets were resuspended to 8 × 10^8^ cells/mL before being allowed to rest for 30 min before experiments. 

### 4.3. Isolation and Culture of Bone Marrow-Derived Megakaryocytes

CD1 and C57BL/6 mice were acquired from Charles River Laboratories and housed in the animal facilities at the University of Manchester, Manchester, UK. All animal work was approved by the University of Manchester and Manchester Metropolitan University Research Ethics committees and carried out under approved home office licences. Mice were anaesthetised using isoflurane and sacrificed by cervical dislocation as per Schedule 1: methods of humane killing.

Femurs were isolated and cut at the knee, and bone marrow was obtained by centrifugation at 2500× *g* for 40 s as previously described by Heib et al., 2021 [64]. Bone marrow was strained through 70–100 µm cell strainers prior to hematopoietic stem and progenitor cell (HSPC) isolation and lineage depletion using an antibody mixture (Lineage depletion panel, 133307, Biolegend) and magnetic beads (CD4 untouched, 11415D, Invitrogen). For MK maturation, HSPCs were incubated in Dulbecco’s Modified Eagle’s Medium (DMEM)GlutaMAX (Fisher Scientific, Leicestershire, UK) complete medium containing thrombopoietin TPO (50 ng/mL) for four days. Differentiated mature MKs were enriched using a BSA density gradient and used directly in transwell migration assays (as described below).

### 4.4. Cell Culture

MEG01 cells were cultured in RPMI media supplemented with 10% (*v*/*v*) foetal calf serum (FCS), 1% Penicillin/Streptomycin, and 2 mM L-glutamine. Cells were kept at 37 °C in a humidified 5% CO_2_ incubator. Every two to three days, adhered cells were removed by scraping, and cells were counted and resuspended in fresh media at a concentration of 1–2 × 10^5^ cells/mL in a vented suspension flask (Sarstedt, Nümbrecht, Germany). Cells were used up to passage 15 and typically between low passages 5–10.

### 4.5. Flow Cytometry

#### 4.5.1. CXCR4 Surface Expression

PRP, MEG01 cells, or primary mouse megakaryocytes were incubated with Pim kinase inhibitors AZD1208, PIM-447 (100 µM in PRP to account for plasma binding [34], otherwise 10 µM), or the vehicle control (0.1% DMSO) for the time stated in the figure legend, before being stained with an antibody raised against the extracellular portion of the CXCR4 (anti-CD184-APC). Analysis was performed using flow cytometry. Data were collected using a MACsQuant Analyser 16 flow cytometer (Miltenyi Biotec, Surrey, UK). The positive percentage was gated using an IgG control, and 10,000 events were counted based on platelet FSC and SSC profiles. For MEG01 cells and primary megakaryocytes, cells that were CD41+ were analysed. 

#### 4.5.2. CD62P Exposure and Fibrinogen Binding

PRP was incubated with Pim kinase inhibitor AZD1208 (100 µM) or the vehicle control (0.1% DMSO) for 10 min prior to stimulation with 1 µg/mL CXCL12 for 20 min at room temperature and incubated with a phycoerythrin (PE)/Cy5 anti-human CD62P (P-selectin) or fluorescein isothiocyanate (FITC)-labelled anti-fibrinogen antibody. Reactions were stopped after 20 min by dilution in phosphate-buffered saline. Data was collected using a MACsQuant Analyser 16 flow cytometer (Miltenyi Biotec, Surrey, UK). The percentage of positive was gated using an IgG negative control, and 10,000 events were counted based on platelet FSC and SSC profiles. The median fluorescence intensity was collected on that gate. Flow cytometry data were analysed using Flow Logic software 8.7.

### 4.6. Calcium Assays

MEG01 cells were added to black 96-well plates and loaded with Fluo-4 AM (Invitrogen) according to the manufacturer’s instructions for 90 min. Fluo-4 AM-loaded MEG01 cells were pre-treated with AZD1208 (10 µM) or the vehicle (0.1% DMSO) for 30 min prior to stimulation with CXCL12 (250 ng/mL), and calcium mobilisation was determined via the measurement of fluorescence using a GloMax® Explorer plate reader (Promega, Southampton, UK). Fluorescence measurements with excitation at 475 nm and emission between 500–550 nm were recorded over a 10 min period. Calcium traces were plotted and the area under the curve was calculated. 

### 4.7. Transwell Migration Assay

Mature primary megakaryocytes were washed and resuspended in serum-free media with AZD1208 (10 µM) or the vehicle (0.1% DMSO) for 30 min before being placed on Corning® Transwell® membrane inserts (Merck Life Science UK Limited, Dorset, UK) with 8 µm pores, with 300 ng/mL CXCL12 in a serum-free medium before being allowed to migrate for 5 h. The top of the membrane inserts was washed to remove any non-migrated cells, and the membrane was stained with DAPI and mounted. Inserts were imaged using a CELENA S Digital Imaging System (Labtech, UK), and the number of cells was counted using ImageJ (Fiji, version 2.15.1) [65].

### 4.8. Live Cell migration/Motility Assay

MEG01 cells were plated in 24-well plates and allowed to attach for three days before non-adherent cells were washed off. Cells were serum-starved overnight in serum-free media, then cells were treated with AZD1208 (10 µM) or the vehicle (0.1% DMSO) for 24 h in the presence of 100 ng/mL CXCL12. Images were acquired at 37 °C and 5% CO_2_ using a Holomonitor M4 live cell imaging system (Phase Holographic Imaging, PHI, Lund, Sweden). Data analysis was performed using the HoloMonitor App Suite 3.5.1 (PHI AB, Lund, Sweden) to calculate average cell motility and migration. 

### 4.9. Gene Expression Studies

MEG01 cells were treated with AZD1208 (10 µM) or the vehicle (0.1% DMSO) for 24 h before RNA was extracted using a Norgen Total RNA Purification Plus Kit (GeneFlow, Lichfield, UK). cDNA was synthesised using a QuantiTect Rev. Transcription Kit and qPCR using a QuantiNova SYBR Green PCR kit (both Qiagen, Manchester, UK) with 10 ng cDNA to assess gene expression. Cycling conditions were 95 °C for 3 min, then 40 cycles of 95 °C for 10 s and 62 °C for 30 s. 

All primers were purchased from Sigma Aldrich (Merck, Dorset, UK). Pim-1 was as follows: 5′-3′ TTCTGGCAGGTGCTGGAGGC and 5′ to 3′ GCGGATCCACTCTGGAGGGC. Pim-2 was as follows: 5′ CTTCGCAGGACACCGCCTCA 3′ and 5′ to 3′ AAAGGCCGCTCGAGGACCAG. CXCR4 was 5′ to 3′ TCGTGCCAAAGCTTGTCCCTG and 5′ to 3′ GCGGTAACCAATTCGCGAATAGTGC, as described in [66]. Housekeeping primers GAPDH and RPLPO were as follows: GAPDH 5′ CGGATTTGGTCGTATTGGGCG 3′, 5′ GTCTTCACCACCATGGAGAAGGC 3′, RPLPO 5′ GCAGCAGATCCGCATGTCCC 3′, and 5′ TCCCCCGGATATGAGGCAGCA 3′. Data were analysed using the ΔΔ^CT^ method normalised to RPLPO [67].

### 4.10. Western Blotting 

MEG01 and washed platelet lysates were prepared in a Laemmli sample buffer for separation via SDS-PAGE electrophoresis and then direct analysis using western blotting. Western blotting was performed using standard techniques as described previously [68]. Proteins were detected using fluorophore-conjugated secondary antibodies and visualised using a LI-COR imaging system. Band intensities were quantified using ImageJ (Fiji, version 2.15.1).

### 4.11. Statistical Analysis

All experiments were performed to *n* ≥ 3. Statistical analyses of the data were carried out using GraphPad prism software (Version 9) unless stated otherwise. When comparing two sets of data, a paired, two-tailed Student’s *t*-test (simple) statistical analysis was used. If more than two means were present, significance was determined using a one-way ANOVA followed by post hoc Dunnett’s correction (multiple). Where data are presented as normalised, statistical analysis was performed prior to normalisation and using the non-parametric Wilcoxon signed-rank test. *p* ≤ 0.05 was considered statistically significant. Where researcher bias may have impacted data collection and analysis, samples were blinded by an independent researcher prior to analysis. Unless stated otherwise, values were expressed as mean ± SEM.

## 5. Conclusions

Pim kinase inhibitors can attenuate CXCR4 downstream signalling and functional responses in megakaryocytes, demonstrating that Pim kinase inhibitors may offer an alternative strategy to target CXCR4-mediated thrombosis.

## Data Availability

Data is contained within the article.
